# Molluscs from South America to the World: Who and Where Are They?

**DOI:** 10.3390/biology14111538

**Published:** 2025-11-03

**Authors:** Gustavo Darrigran, Ignacio Agudo-Padrón, Pedro Báez, Carlos Eduardo Belz, Franz Cardoso, Alvar Carranza, Gonzalo A. Collado, Modesto Correoso, María Gabriela Cuezzo, Alejandra A. Fabres, Monica A. Fernandez, Suzete R. Gomes, Diego E. Gutierrez Gregoric, Sergio Letelier, César Lodeiros, Sandra Ludwig, María Cristina Mansur, Janine Oliveira Arruda, Guido Pastorino, Pablo Penchaszadeh, Rodrigo B. Salvador, Sonia Santos, Paula Spotorno, Silvana Thiengo, Teofânia Vidigal, Cristina Damborenea

**Affiliations:** 1 División Zoología Invertebrados, Museo de La Plata, FCNyM, CONICET, Paseo del Bosque s/n, La Plata 1900, Argentina; invasion@fcnym.unlp.edu.ar (G.D.); dieguty@fcnym.unlp.edu.ar (D.E.G.G.); 2 Projeto “Avulsos Malacológicos”, Florianópolis 88010-970, SC, Brazil; ignacioagudo@gmail.com; 3 Centro de Investigación Marina Quintay (CIMARQ), Facultad de Ciencias de la Vida, Universidad Andrés Bello, Quintay 2050000, Chile; projasusbaez@gmail.com; 4 Sociedad Malacológica Chile, SMACH, Santiago 9170022, Chile; alejandra.fabres@gmail.com (A.A.F.); chechulucho@gmail.com (S.L.); 5 Laboratorio de Ecologia Aplicada e Bioinvasões, Centro de Estudos do Mar, Universidade Federal do Paraná, Pontal do Paraná 83255-976, PR, Brazil; belzoceanos@gmail.com; 6 Laboratorio de Biología y Sistemática de Invertebrados Marinos, Departamento Académico de Zoología, Facultad de Ciencias Biológicas, Universidad Nacional Mayor de San Marcos, Lima 150101, Peru; fcardosop@unmsm.edu.pe; 7 Departamento de Ecología y Gestión Ambiental, Centro Universitario Regional Este (CURE), Sede Maldonado, Universidad de la República, Maldonado 20100, Uruguay; alvar.carranza@gmail.com; 8 Departamento de Ciencias Básicas, Facultad de Ciencias, Universidad del Bío-Bío, Av. Andrés Bello 720, Chillán 3780000, Chile; gcollado@ubiobio.cl; 9 Departamento Ciencias de la Tierra y la Construcción, Universidad de la Fuerzas Armadas ESPE, Av. General Rumiñahui s/n, Sangolquí 171103, Ecuador; mcorreoso@espe.edu.ec; 10 Instituto de Biodiversidad Neotropical (Conicet-Facultad de Ciencias Naturales UNT), Cúpulas de Horco Molle, Tucumán 4105, Argentina; gcuezzo@webmail.unt.edu.ar; 11 Department of Biological Sciences, Auburn University, Auburn, AL 36849, USA; 12 Laboratório de Referência Nacional para Esquistossomose-Malacologia, Instituto Oswaldo Cruz, Fiocruz, Av. Brasil 4365, Manguinhos, Rio de Janeiro 21040-900, RJ, Brazil; ammonfernandez@gmail.com (M.A.F.); suzete.gomes@ioc.fiocurz.br (S.R.G.); scarvalhothiengo@gmail.com (S.T.); 13 Departamento de Acuicultura, Pesca y Recursos Renovables, Facultad de Acuicultura y Ciencias del Mar, Universidad Técnica de Manabí, Bahía de Caráquez EC131450, Ecuador; cesarlodeirosseijo@yahoo.es; 14 Instituto Oceanográfico de Venezuela, Universidad de Oriente, Cumaná 6101, Sucre, Venezuela; 15 Grupo Integrado de Aquicultura e Estudos Ambientais (GIA), Universidade Federal do Paraná, Curitiba 80060-150, PR, Brazil; sandludwig@gmail.com; 16 Grupo de Pesquisa CNPq, Biodiversidade de Moluscos, Continentais, Museu de Ciências Naturais, Rua Dr. Salvador França, 1427, Porto Alegre 90690-000, RS, Brazil; mcrismansur@gmail.com; 17 Museu de Ciências Naturais do Rio Grande do Sul, Avenue Dr. Salvador França, 1427, Porto Alegre 90690-000, RS, Brazil; arrudajo@gmail.com; 18 Laboratorio de Ecosistemas Marinos, Museo Argentino de Ciencias Naturales “Bernardino Rivadavia” CONICET, Av. Angel Gallardo 470, Ciudad Autónoma de Buenos Aires 1405, Argentina; gpastorino@macn.gov.ar (G.P.); pablopench@gmail.com (P.P.); 19 Finnish Museum of Natural History, University of Helsinki, Pohjoinen Rautatiekatu 13, 00100 Helsinki, Finland; salvador.rodrigo.b@gmail.com; 20 Laboratório de Malacología Límnoca e Terrestre y Programa de Pós-Graduação em Ecologia e Evolução (PPGEE), Departamento de Zoologia, Universidade do Estado do Rio de Janeiro (UERJ), Rio de Janeiro 20550-900, RJ, Brazil; malacosonia@gmail.com; 21 Centro de Ciências Computacionais and Instituto de Oceanografia, Universidade Federal do Rio Grande—FURG, Av. Itália Km 8, Rio Grande 96207-900, RS, Brazil; paula.spotorno@gmail.com; 22 Laboratório de Malacologia e Sistemática Molecular, Departamento de Zoologia, Instituto de Ciências Biológicas, Lelf: Laboratório de Estudos de Limnoperna Fortunei, Centro de Pesquisas Hidráulicas, Universidade Federal de Minas Gerais, Belo Horizonte 31270-901, MG, Brazil; teofania.vidigal@gmail.com

**Keywords:** invasive species, freshwater, land, marine, climate change, globalization, unintentional introduction, intentional introduction

## Abstract

**Simple Summary:**

While the threat of climate change increases, biodiversity is also being affected by another problem, the introduction of non-native species that are resistant to environmental disturbances. Some of these non-native species can become invasive and have negative impacts on native ecosystems, public health, and the economy. In this context, society, government, and the academic community—each according to its role—must manage such species, which is a process involving prevention, detection, and control. In this work, the researchers who compose eMIAS (South American Invasive Mollusks Specialists) focus on compiling a reference work for the 29 mollusc species that are native to South America and that have been introduced to other continents. Among those 29 species, 10 are marine, 10 are freshwater, and 9 are terrestrial. These introductions, over the past five decades, have been facilitated by an increase in global trade as well as in environmental degradation in many areas (e.g., climate change, urbanization).

**Abstract:**

Ecosystems and biodiversity around the globe face multiple threats, including climate change and invasive species. Non-native species are known for their resilience to disturbances and their ability to thrive more successfully than native species in urbanized or otherwise disturbed areas, and some of them can become invasive. It is a complex challenge to detect, manage, and control such species, which require coordinated efforts from society, government, and the academic community. In this study, the eMIAS (South American Invasive Mollusks Specialists) research group (27 experts from seven South American countries) aim to provide foundational knowledge for management of these species. We compiled and synthesized information on the mollusc species that are native to South America and that have been introduced to other regions of the world. A total of 29 species were detected, including 10 marine, 10 freshwater, and 9 terrestrial. For each species, the area of origin, date and place of introduction, and current distribution were determined. We could determine that (1) most of these introductions have occurred in connection with globalization processes, such as an increase in trade. (2) The potential source regions of those 29 species are also areas that received non-native species from elsewhere (e.g., Europe, Asia). (3) Regions where species introductions have taken place are subject to the impacts of climate change and/or urbanization.

## 1. Introduction

Global climate change, land use and habitat degradation, as well as invasive species, all threaten the integrity of ecosystems [1]. The pace of global change over the past 50 years is unprecedented in human history. The combination of the multiple drivers of change in nature—shaped by societal values and behaviours (e.g., human population dynamics, technological innovations, and patterns of production, trade and consumption)—promotes and accelerates these changes, though in different manners and rates across countries [2]. These developments are so significant that many researchers refer to the current period as the Anthropocene [3] or, less commonly, the Capitalocene [4,5]. Conceptual debates aside, land use (urbanization and agricultural intensification) is profoundly transforming environments, causing habitat loss, altering physicochemical conditions, and facilitating the establishment of non-native species [6,7]. Ultimately, that affects organisms’ distribution and functionality within ecosystems [6].

Non-native species, when they become invasive, represent one of the most critical environmental challenges of our time. Invasive species can impact ecosystems and/or socioeconomic systems (i.e., nature’s contributions to people and quality of life) [8,9]. Invasive non-native species are considered one of the main drivers of biodiversity loss [10] and also impose multi-billion-dollar socioeconomic costs [11,12,13]. When managing invasive species, it is essential to consider not only environmental impacts but also the historical and cultural dimensions of the societies involved, which are often overlooked [14]. Thus, effective management of bioinvasions requires avoiding conflating environmental impacts with societal or economic ones [15].

Major cities and ports serve as gateways for the introduction of non-native species [16], which is now happening at higher rates due to global trade and human movement [17,18]. More specifically, European colonialism in the 19th century and the increase in commerce during the 20th and 21st centuries have intensified the movement and establishment of species [19]. Presently, climate change is creating environments that are increasingly receptive to new invasions by making them inhabitable by a wider array of non-native species. Biotic resistance tends to be lower in altered environments, which facilitates the process of colonization by non-native species [20], particularly when environmental conditions (e.g., climate) are similar between the original and new habitat [21]. Life history traits of the species involved (e.g., reproductive strategy and rate, feeding habits, dispersal strategies) are related to their “invasibility” and can partly explain their invasion history [7,21]. The remainder of the explanation is more mundane, including questions of vectors, pathways, and propagule pressure [22], though tracing those is typically not straightforward.

Within the framework of species movement around the globe, molluscs represent an interesting case. The phylum is the second most diverse, with representatives widely distributed globally across aquatic and terrestrial environments. Societies have historically used—and still use—molluscs for food and cultural purposes (from ornaments to pets), and people have carried these animals around both intentionally and unwittingly. Like most animals, molluscs can also act as vectors for pathogens, some of them of critical medical importance [23]. Naturally, some mollusc species, typically bivalves and gastropods, can become invasive.

Managing these species involves challenges related to detection, control, and coordination among society, governments, and the scientific community [10]. Our research consortium, eMIAS (South American Invasive Molluscs Specialists), was created to study non-native and alien species in South America. It has been producing reference papers on the topic for the past five years [24,25,26], identifying vectors and pathways of introduction in the continent, assessing the impacts of some invaders, and also examining native South American species transplanted to new areas within the continent itself [25,26,27]. In this context, the present study goes beyond the borders to compile and synthesize information on South American molluscs that have been introduced to other continents. In this study, we address a knowledge gap by compiling and analyzing the available information on South American native molluscs recorded outside their native range.

## 2. Materials and Methods

According to the International Union for Conservation of Nature (IUCN), invasive species are species that have been introduced by human action to areas outside their natural range (non-native species) that have negative impacts on native biodiversity, or negative effects on ecosystem services and human economy [8]. In this study, species are classified as non-native when their occurrence in a specific geographic area is directly attributed to human action [24].

A network of malacological and taxonomic experts from South America (Argentina, Brazil, Chile, Ecuador, Peru, Uruguay, and Venezuela), all members of the eMIAS research group (South American Invasive Molluscs Specialists; https://emiasgroup.wixsite.com/emias; accessed on 30 October 2025 ), collaboratively reviewed published and unpublished records of Mollusc Species Native to South America and Non-Native Elsewhere (M-NSA-NNE). The group systematically compiled and synthesized information for each M-NSA-NNE, including taxonomic identity, native range, first record and year of introduction or detection outside South America, known introduction vectors, ecological impacts, and effects on humans.

The initial list of candidate taxa was filtered to include only species supported by reliable and verifiable data, specifically those reported in peer-reviewed literature with a significant impact factor and endorsed by eMIAS specialists across different taxa and regions. The final list of species was then summarized in three tables, each corresponding to a different environment (marine, freshwater, and terrestrial). For each species, the following data were standardized and cross-validated among experts: (1) systematic classification and species identification, following MolluscaBase [28]; (2) native distribution in South America classified as ecoregion following Olson et al. [29] for terrestrial, Spalding et al. [30] for marine, and Abell et al. [31] for freshwater. Numerical codes for ecoregion are provided in Supplementary Materials Figures S1–S3; (3) non-native distribution, classified into biogeographical realms and subrealms as defined by One Earth [32], including location and year of first detection outside South America, subsequent establishment sites, and relevant bibliographic references; (4) introduction mechanisms, including pathway categories and subcategories as proposed by Faulkner et al. [33], differentiate natural dispersal from human-mediated dispersal; this represents the first classification of non-native species presented in Table 1, Table 2 and Table 3; (5) remarks on invasiveness potential, based on ecological and biological traits (e.g., habitat type, environmental tolerance, mobility, feeding strategy, association with anthropogenic environments, known impacts, economic effects, host status for humans or domestic animals, reproductive strategy and frequency, developmental mechanisms, parental care, dispersal capacity, and fertilization strategy; (Table S1). Due to the limited knowledge of the biology of many native South American molluscs, the biological characteristics considered in this study were analyzed qualitatively using Atlas.ti software (25.0.1).

Additionally, the native distribution of M-NSA-NNE species was compared with the hotspot regions of non-native mollusk introductions identified by Darrigran et al. [24] to evaluate potential geographical overlap between source areas and recipient regions. The Subtropical Atlantic, Northern Andes, Central Andes, and Southern Andes zone are hotspot areas [24] that are characterized by a high degree of urbanization, including large cities, passenger and cargo airports, and seaports.

Data visualization included the construction of Sankey diagrams and word clouds, both generated using the free version of Atlas.ti software (25.0.1).

## 3. Results

### 3.1. Native Mollusk Species Detected Outside of South America

A total of 31 M-NSA-NNE species were detected (Table 1, Table 2 and Table 3), including nine terrestrial, ten freshwater, and twelve marine species. Two marine gastropods, *Onchidella marginata* (Couthouy, 1852) and *Pareuthria fuscata* (Brugiere, 1789), were introduced through natural dispersal (rafting on sea kelps) without human intervention. For this reason, these species were not considered as non-native and were excluded from further analyses. Among the remaining species, 21 were gastropods, 5 were bivalves, and 3 were polyplacophores.

The following exceptions were detected:

1. *Pomacea glauca* (Linnaeus, 1758) a gastropod native to South America and non-native to the Dominican Republic [111], was classified as Least Concern by Pastorino and Darrigan [112] and, due to this status and the lack of conservation concern, was not included in the final list of species in Table 2 (S. Thiengo, personal communication).
2. *Naesiotus quitensis* (L. Pfeiffer, 1848) (=*Bulimus quitensis* L. Pfeiffer, 1848), a terrestrial gastropod native to South America, was erroneously cited for Spain due to the presence of empty shells found in urban parks in Madrid [113]). This gastropod is native to Ecuador, where it is used in a traditional national dish called “ceviche de churos”, in which the snails are cooked and seasoned. Ecuadorians have actively exported this dish every year between October and December, typically with dead and cooked individuals of *N*. *quitensis* (M. Correoso, personal communication). Therefore, it is an accidental transport of shells via food exports.

### 3.2. Areas of Origin of the Species

**Figure 1:**
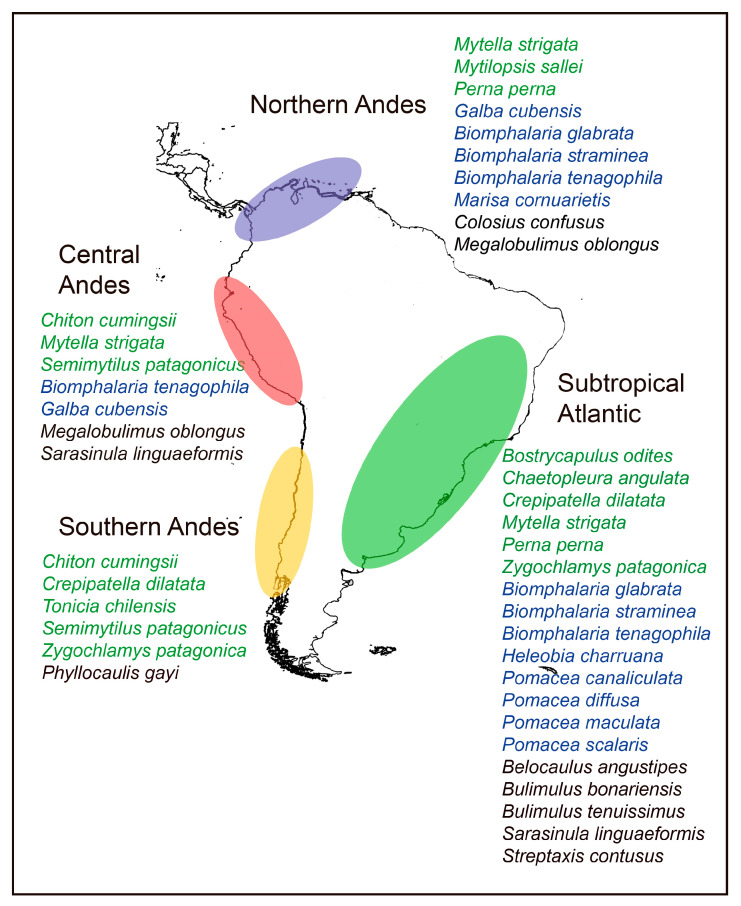
Native South American mollusc species introduced to other regions (M-NSA-NNE), coinciding with the hotspot areas for introduced mollusc species identified by Darrigran et al. [24]. Marine species in green; freshwater species in blue; terrestrial species in black.

**Figure 2:**
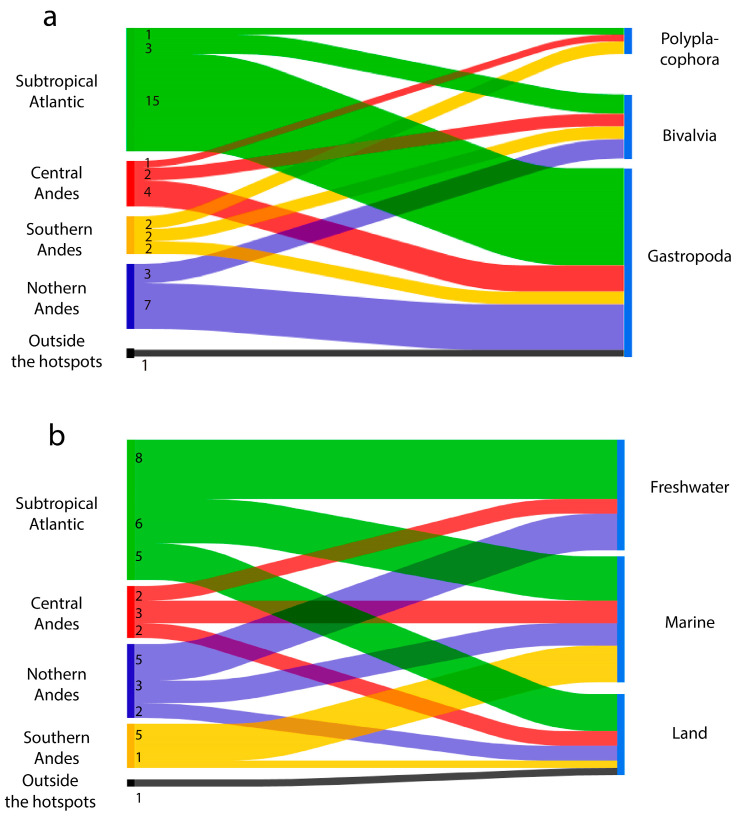
Relationship between hotspot areas determined by Darrigran et al. [24], and the class (**a**), and environment type (**b**) of M-NSA-NNE species. Numbers on the right indicate the total number of species per hotspot area.

The presence of the 29 M-NSA-NNE overlaps with the four areas of South America identified by Darrigran et al. [24] (Figure 1), where the highest number of non-native molluscs has been recorded since 1970. Only *Tamayoa decolorate* was registered in the Amazonian subrealm, with no connections to the previously mentioned areas. The area where M-NSA-NNE species are most diverse is the Subtropical Atlantic, with a total of 19 species, followed by the Northern Andes with 10 species (Figure 2). In the Subtropical Atlantic, the greatest species richness belongs to gastropods (15). In the four areas considered, the number of bivalves and polyplacophores species varies between one and three.

**Figure 3:**
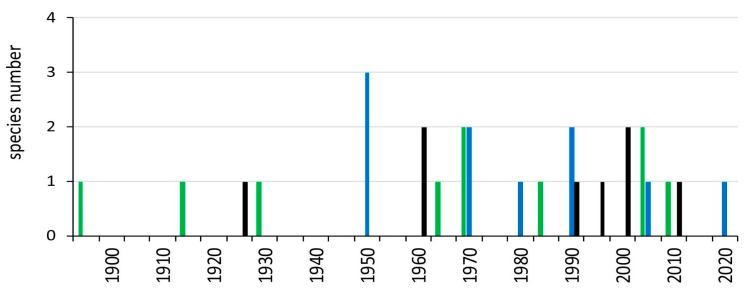
Date of first record occurrence of M-NSA-NNE mollusc species outside of South America. Records are coloured by habitat: green for marine, blue for freshwater, black for terrestrial.

The largest number of M-NSA-NNE species recorded are marine (Figure 3). The greatest species richness is found in the Subtropical Atlantic region, with six species, followed by the Southern and Northern Andes, each with five species. Freshwater species rank next in richness, with eight species recorded in the Subtropical Atlantic region, and five in the Northern Andes. Finally, five terrestrial species are present in the Subtropical Atlantic, two in the Northern and Central Andes, and only one species in the Southern Andes. One terrestrial species is not related with these zones.

### 3.3. Detection Outside of South America

Although there are some records of M-NSA-NNE species prior to 1950 (e.g., *Chetopleura angulata* in the 19th century; *Perna perna* in 1917; *Phyllocaulis gayi* in 1925; *Semimytilus patagonicus*, as *S*. *algosus*, in 1928), it was not until the 1950s that freshwater species were first detected, and the presence of M-NSA-NNE species began to become permanent (Figure 3). From 1980 onwards, the number of new detections remained practically constant.

### 3.4. Introduction Mechanisms

**Figure 4:**
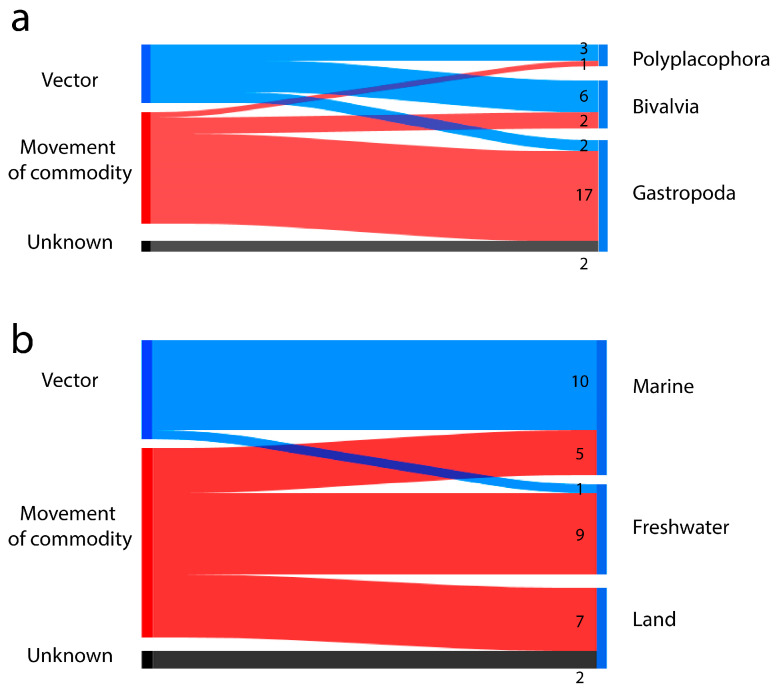
Mechanisms of introduction for M-NSA-NNE species according to Falkner et al. [33], Shown by (**a**), mollusc class and (**b**), environment. Numbers on the right indicate the total number of species per mechanism.

The introduction mechanism that contributed most to the dispersal of M-NSA-NNE species was movement of commodity, accounting for a total of 20 species, of which 17 were gastropods, 2 bivalves, and 1 polyplacophore. The second most important mechanism were vectors, with a total of eleven species, of which six were bivalves, three polyplacophorans, and two gastropods (Figure 4a).

Most freshwater and terrestrial M-NSA-NNE species were transported by movement of commodity (16 species). Only one freshwater species was transported by vectors, while the transport mechanism remains unknown for the two terrestrial species (Figure 4b).

**Figure 5:**
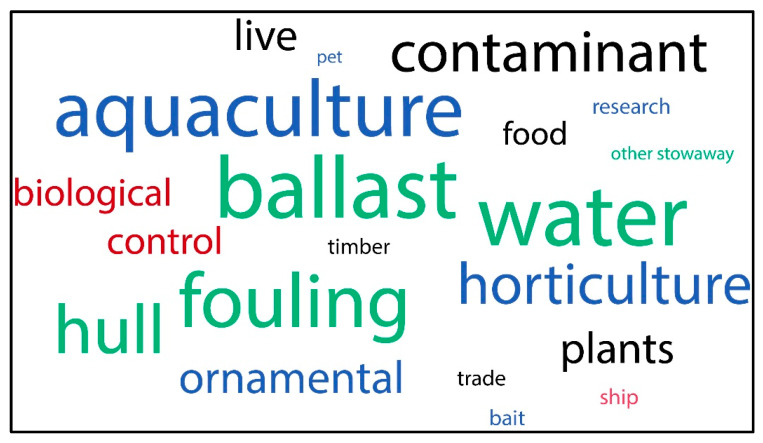
Frequency of pathway subcategories for M-NSA-NNE species introduction according to Falkner et al. [33]. Introduction mechanisms are colour coded: movement of commodity release in red, escape in blue, contaminant in black, mechanism of introduction vector stowaway in green.

In the marine environment, most of the M-NSA-NNE species were introduced by vessels as a vector (10 species), either by ballast water, fouling on hulls, chains, anchors, or similar means. The remaining five species were introduced through movement of merchandise associated with aquaculture, food, and live bait (Figure 4b and Figure 5).

### 3.5. Relationship Between Biological Characteristics and the Mechanisms of Introduction, Pathway Categories, and Subcategories

**Figure 6:**
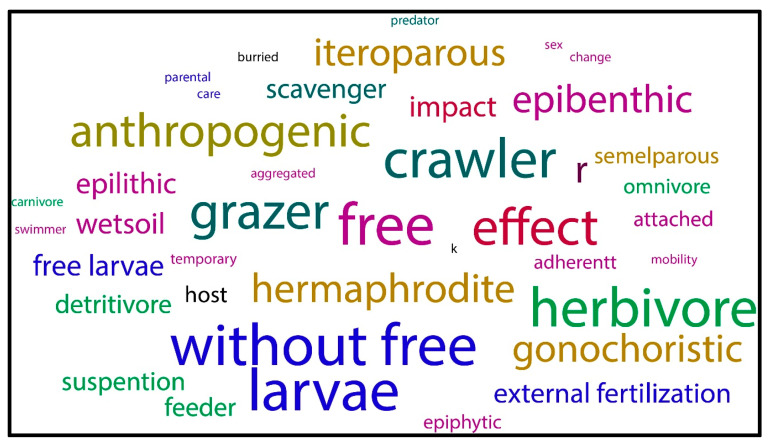
Representation of the frequency of biological characteristics of M-NSA-NNE species, and their impact or effect and whether it is linked to human activity.

The literature describes that M-NSA-NNE species are free-living. The predominant mechanism of introduction for M-NSA-NNE species is by movement of commodity (Figure 4 and Figure 5), which may occur through contaminants, escape, or intentional release. When analyzing the consequences caused by these species, the term “effect” referring to their impact on human societies and economies predominates over the term “impact”, referring to consequences caused on the natural environment (Figure 6). Furthermore, many of the species considered are closely linked to anthropogenic activities.

Regarding feeding style, most invasive species are herbivorous. A smaller number are scavenger species. Grazers are also more numerous than omnivorous, detritivore, and suspension feeders (Figure 6).

Regarding reproductive style, there is a predominance of M-NSA-NNE species with direct development, meaning they do not have a free-swimming larval stage. The number of gonochoristic and hermaphrodite species is similar. The prevalence of the r-strategy is notable, along with a marked tendency towards iteroparity.

The predominant lifestyles among M-NSA-NNE species are free living and crawling. In contrast, the number of species with a buried lifestyle is lower than the combined total of the species with epifaunal lifestyles including epibenthic, epilythic, adherent forms.

### 3.6. Areas of Introduction

**Figure 7:**
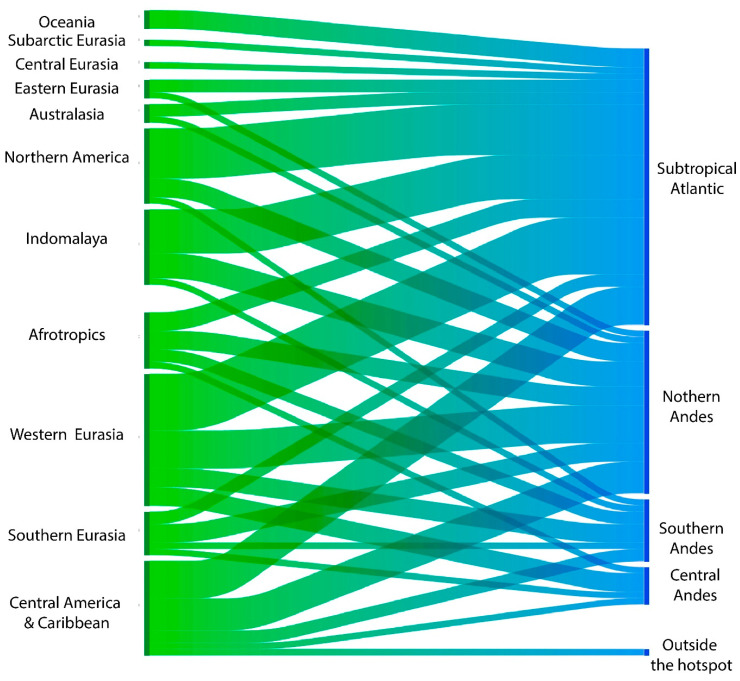
Relationship between the major biogeographical realms [32] with introduced M-NSA-NNE species and the areas of South America described as hotspots in Darrigran et al. [24].

M-NSA-NNE species have been introduced to 85% of the biogeographical realms worldwide. Notably, species originating from the Subtropical Atlantic Zone of South America were introduced into all of these realms. In comparison, 72% of realms contain species from the Northern Andes, 57% from the Southern Andes, and 36% from the Central Andes (Figure 7).

**Figure 8:**
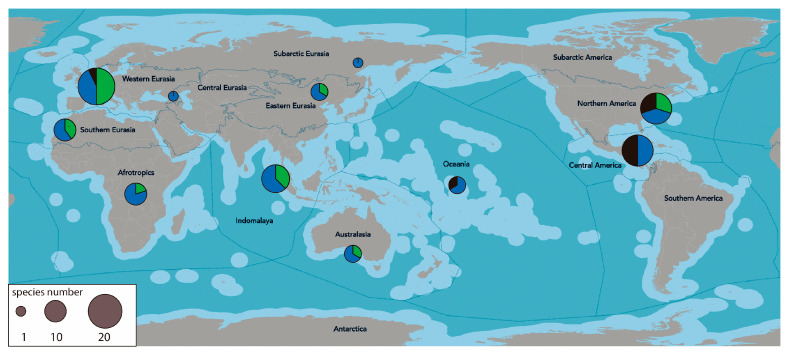
Frequency distribution of M-NSA-NNE species across the major biogeographic realms [32]. Circle sizes represent the number of species per realm; species are colour-coded by habitat: blue for freshwater, green for marine, and black for terrestrial.

The kingdom with the highest number of NNE-MSA species is Europe (Western Eurasia), followed by the Northern American kingdom. In contrast, the kingdoms with the fewest NNE-MSA species are Subarctic Eurasia and Central Eurasia (Figure 8).

Freshwater NNE-MSA species are found in 100% of the kingdoms considered, while marine species are found in 63%, and terrestrial species in 36% (Figure 8). Terrestrial species are restricted to the Central and North American kingdoms, specifically within Southeast American Savannahs and Woodlands subkingdom. Finally, the lowest number of NNE-MSA species are found in Oceania and Western Asia.

## 4. Discussion

This study highlights the mollusc species that are native to South America, and have been introduced (i.e., are non-native) in other continents (M-NSA-NNE), including both aquatic and terrestrial forms. Our findings show that these species have been introduced into all biogeographic realms except Antarctica and Subarctic America. Carneiro et al. [19] argued that there is a regional pattern in the origin of invasive species, with the Palearctic and Oriental regions being the main exporters, while the Nearctic and Neotropical regions primarily act as recipients. Still, this study records 29 M-NSA-NNE species that have been introduced elsewhere.

In general, the number of non-native species continues to rise globally and across taxa, with no signs of saturation. Increasing rates of ‘first records’ have been observed over time, and efforts to prevent and mitigate invasions so far have proven insufficient [17]. Human action has drastically reshaped landscapes worldwide. A common trait of non-native species is their tolerance to anthropogenic disturbances and their ability to thrive in urban landscapes, where native species are typically less abundant [6]. Presently, 56% of the global population lives in densely populated urban and peri-urban areas that act as “hotspots” for species introductions, with high abundance of non-native species [114]. That tendency is also observed within South America, where four major urbanized zones have the highest number of non-native and invasive molluscs in the continent [24]. The present study further reveals that these same four zones are likely the origin points for M-NSA-NNE species introduced to other continents, with the Subtropical Atlantic zone standing out among them and hosting 85% of the detected M-NSA-NNE species.

It is important to highlight that taxonomic and distributional knowledge of many mollusc species in South America remains limited, sometimes making it difficult to classify them as native or non-native. For instance, Robinson [11] mentions 15 freshwater and 47 terrestrial mollusc species introduced into the United States from Latin America, for which their natural distribution is unknown.

Records of M-NSA-NNE species date back to the early 20th century (e.g., *Chaetopleura angulate* and *Perna perna* in 1917; *Phyllocaulis gayi* in 1925; *Semimytilus patagonicus* in 1928). However, from 1950 onwards—and especially from 1980 onwards—their spread vastly intensified in tandem with global trade and climate change [115,116,117,118] and the likelihood of both intentional and accidental transport of species [117]. Notably, many of these M-NSA-NNE species introductions would likely not succeed if the ecosystems where they arrived had not already been impacted by climate change [9].

While elucidating vectors and pathways are important for assessing invasion risk, there are also biotic and abiotic filters (e.g., biotic resistance, environmental compatibility) that influence predictions [119]. Our study highlights the importance of the movement of commodities, once again underscoring the significant role of global trade in the introduction of these species. There has been a steady increase and continued recording of M-NSA–NNE species since 1980, and they are now present in nearly all biogeographic realms. Understanding their traits and life histories is also important, and some M-NSA-NNE species indeed possess traits that favour colonization (e.g., generalist diet, iteroparity, r-strategy). Early knowledge of these species could enable early response and improve their effectiveness, perhaps even to avoid their establishment in new environments [13].

Other decisive factors in species introductions include the invasibility of the recipient environment [21] and the political, social, and economic context [19]. The literature shows that the effects of the introduced species on societies often outweigh the environmental impacts and thus, the generation of knowledge about non-native and invasive species is more closely linked to socioeconomic relevance than to their environmental consequences. Similarly, while invasive species are considered a driver of biodiversity loss [10], there is also a large focus on how biodiversity loss affects human societies rather than how it affects global ecosystems [120]. It is evident that the support for research on invasive species affecting humans is much greater than for the research on their environmental impacts. This stems from an anthropocentric perspective, creating a financial bias toward oriented research that entails negative operational and economic costs, to the detriment of studies on the species-specific characteristics of native species in general.

It is essential for society to be capable of identifying whether a non-native is indeed invasive, understand any impacts early on, and provide means to (1) highlight management shortcomings in early detection and response, and (2) use novel resources (e.g., artificial intelligence) to facilitate early detection and indicate potential pathways for solutions [13]. To that end, it is crucial to integrate ecological, political, and economic research with programmes to raise public awareness about non-native and invasive species through both local and global educational initiatives. In this regard, increasing taxonomic knowledge is essential to accurately identify species. The case of *Bulimulus* tree snails, some of which can affect crops, illustrates this gap, as these snails are difficult to distinguish, and some species may actually represent groups of cryptic taxa.

Carlton and Ruiz [121] already emphasized the importance of understanding the mechanisms by which species are transported via human activity and recognizing the diversity of species that could be mobilized. In line with this, the findings of this study provide foundational knowledge for environmental managers in both the public and private sectors, as well as for conservationists and policymakers, to support monitoring [122,123,124,125] and management efforts [10,23]. Furthermore, to carry out this activity successfully over time, there must be knowledge sharing and open dialogue with society. A key area is to improve the perception of urban areas as valuable spaces for biodiversity conservation, complementing studies conducted in more pristine and protected areas. Meanwhile, it is also crucial that management efforts consider key elements that contribute to the quality of life of local populations [114]. Finally, incorporating this knowledge into both formal and informal education is essential for creating a more informed society that can demand and guide actions of managers and policies, thereby contributing to biodiversity conservation in a context of globalization and climate change.

## 5. Conclusions

Through the collaboration of the eMIAS (South American Invasive Mollusc Specialists) group, a comprehensive and up-to-date bibliographic compilation of native South American mollusc species that have established populations on other continents was achieved. A total of 29 M-NSA-NNE species were identified, including 10 marine species, 10 freshwater species, and 9 terrestrial species. No differences were found in the number of M-NSA-NNE species according to their habitat.

Based on three global factors identified as promoting bioinvasion, in general, and the spread of mollusc species in particular—climate change, globalization, and the presence of large cities—this paper highlights two key points:

The existing literature focuses more on the socioeconomic “effects” than on the environmental “impacts” caused by M-NSA-NNE species, indicating an anthropocentric bias in the funding of research on this topic.

There are four regions in South America where these native species are distributed and from which they could potentially spread as M-NSA-NNE to other locations worldwide. These regions are equipped with large cities, ports, and airports for both cargo and passenger transport. Notably, these regions coincide with those identified as entry points for non-native/invasive mollusc species in South America.

This study establishes trends in the mechanisms by which M-NSA-NNE species are transported, and identifies the diversity of species that have been mobilized. Accordingly, the findings provide a knowledge baseline for environmental managers, conservationists, and policymakers to support monitoring and management efforts.

## Figures and Tables

**Table 1:** Marine mollusc species native to America del Sur and non-native in other areas. Species and species ID following MolluscaBase [28]; place and date of entry from first record, localities where the species has been registered, introduction mechanism, pathway category, and pathway subcategory after Falkner et al. [33]; for ecoregions reference number see Darrigran et al. [24] and Figure S1.

	Species and Species ID	Ecoregions in South America	Place and Date of Entry and Reference	Introduction Mechanism, Pathway Category, and Subcategory	Impacts/Effects/Remarks
**Polyplacophora | Chitonida | Chaetopleuridae**					
	*Chaetopleura angulata* (Spengler, 1797) https://www.molluscabase.org/aphia.php?p=taxdetails&id=848025, accessed on 1 April 2025	18, 19, 21, 22	Atlantic coast of the Iberian Peninsula, XIX century or before [34,35,36]; Bay of Biscay and south of Portugal [36] to the Straits of Gibraltar [35,37,38]; Cantabrian [39] and Asturias [40]	Vector, stowaway, ship excluding ballast water and hull fouling [34] Ballast water? [41]	**Impacts: -** **Effects: -** **Remarks**: Possibly transported by Spanish or Portuguese merchant vessels to the Iberian coasts, due to the animal’s ability to climb anchor chains rather rapidly [34].
**Polyplacophora | Chitonida | Chitonidae**					
	*Chiton cumingsii* (Frembly, 1827) https://www.molluscabase.org/aphia.php?p=taxdetails&id=386774, accessed on 1 April 2025	9–14	Las Palmas Port, Canary Islands, 28°06′ N, 15°25′ W, 2012 [42,43]	Vector, stowaway, hull fouling [42]	**Impacts: -** **Effects: -** **Remarks: -**
	*Tonicia chilensis* (Frembly, 1827) https://www.molluscabase.org/aphia.php?p=taxdetails&id=386329, accessed on 1 April 2025	12–14, 17	Asturias and Galicia, around 1970 (Eo estuary, 43°28′ N, 7°03′ W; Sado estuary, 1985, 43°28′ N, 7°03′ W, Aviles Port 43°33′ N, 5.55′ W) [42]	Vector, stowaway, hull fouling, ballast water (?) Movement of commodity, escape, aquaculture (?) [41,42]	**Impacts: -** **Effects: -** **Remarks**: The northern ecotype of *T. chilensis* inhabits the coast of central Chile (~33–39°S), whereas the southern is found from Puerto Mont (~41°S) to Tierra del Fuego (~53°S).
**Gastropoda | Littorinimorpha | Calyptraeidae**					
	*Crepipatella dilatata* (Lamarck, 1822) https://www.molluscabase.org/aphia.php?p=taxdetails&id=234137, accessed on 1 April 2025	12–19	Galician in Ría de Aldán, 2005; north coast of Iberian Peninsula, 2018; in Ebro delta (Mediterranean), 2014 [44]	Movement of commodity, escape, aquaculture [44]	**Impacts: -** **Effects:** Fouling (at a shellfish treatment plant in Palmeiras [45]). **Remarks**: The name *Crepipatella dilatata* was commonly used in Peru [46], but according to Veliz et al. [47], it refers to *C. peruviana.*
	*Bostrycapulus odites* (Collin, 2005) https://www.molluscabase.org/aphia.php?p=taxdetails&id=457048, accessed on 1 April 2025	18, 19, 21, 22	Alicante Harbour, 1970s [48]	Vector, stowaway, ballast water [49]	**Impacts: -** **Effects: -** **Remarks**: Referred to as *Crepidula calyptraeformis* and *C. aculeata* [48]. Common and widely distributed in Alicante Harbour in 2002 and 2007, but has not expanded outside the harbour [48].
**Bivalvia | Mytilida | Dreissenidae**					
	*Mytilopsis sallei* (Récluz, 1849) https://www.molluscabase.org/aphia.php?p=taxdetails&id=397147, accessed on 1 April 2025	1, 7, 4	India (approx. 1967); Japan, 1974; Taiwan, 1977; Hong Kong, approx. 1980; Singapore and Malaysia, 1984?; China, approx. 1990; Thailand, between 1990 and 2000; Australia (1999); Egypt (2006); Philippines, 2008; Israel, approx. 2009 [50,51,52,53,54,55,56,57]	Vector, stowaway, hull fouling	**Impacts:** Displacement of native species. **Effects:** On aquaculture (of shrimp, by water clarification). **Remarks**: Highly opportunistic species and can survive under extreme and wide-ranging conditions.
**Bivalvia | Mytilida | Mytilidae**					
	*Mytella strigata* Hanley, 1843 [= *Mytella charruana* (d’Orbigny, 1846)] https://www.molluscabase.org/aphia.php?p=taxdetails&id=1458663, accessed on 10 April 2025	1–4, 7–10, 19, 21–23, 25–27	Florida, 2006; Philippines, 2014; Singapore 2016; Thailand, 2018; Cochin and Mannar Golf India and Sri Lanka, 2019; southwest coast of Taiwan, 2021; Beibu Gulf, 2021; Hong Kong, 2022 [58,59]	Vector, stowaway, ballast water	**Impacts:** Displacement of native species (compete with native species for resources, alter habitat structures, and disrupt local food chains). **Effects:** On aquaculture (of shrimp, by water clarification) [59]. **Remarks**: Often thriving in new environments without natural predators or competitors [60]. For native distribution, see Valentich-Scott et al. [61].
	*Perna perna* (Linnaeus, 1758) https://www.molluscabase.org/aphia.php?p=taxdetails&id=140483, accessed on 12 April 2025	1, 4, 7, 15, 19, 21–23	South India and Sri Lanka, approx. 1917 [62]; Israel, 1965 [63]; Mozambique, 1970 [64]; Arkansas, Texas, 1990; Golf of Mexico, approx. 1990 [65]; Portugal, 2011 [66]; from Ludertz to Gibraltar Strait, Tunisia, 2020; Israel, 2020 [64]; Yemen [67]	Vector, movement of commodity, stowaway, escape, hull fouling, ballast water, aquaculture [64]	**Impacts: -** **Effects:** Fouling (on navigation buoys and pipelines); food resource. **Remarks: -**
	*Semimytilus patagonicus* (Hanley, 1843) https://www.molluscabase.org/aphia.php?p=taxdetails&id=1518612, accessed on 15 April 2025	9–13, 18, 26, 28	Namibia, Walvis Bay 1928–1929; Northern Angola, 1969 Moçâmedes [68] to Elands Bay Northern South Africa, 2009 or sometime earlier [69]	Vector, stowaway, hull fouling? movement of commodity, escape, aquaculture?	**Impacts: -** **Effects:** Potential on aquaculture. **Remarks**: Originally described from Río Negro, Northern Patagonia, Argentina, where the population is no longer present. Alternatively, the name *S. algosus* has been used instead, particularly in Chile and Africa. Recently, Signorelli and Pastorino [70] resurrected the older name, *Semimytilus patagonicus*. For native distribution see [61]
**Bivalvia | Pectinida | Pectinidae**					
	*Zygochlamys patagonica* (P. P. King, 1832) https://www.molluscabase.org/aphia.php?p=taxdetails&id=236717, accessed on 1 April 2025	14–21	Mediterranean Sea, 1985 [71]	Movement of commodity, escape, live food and live bait [72]	**Impacts: -** **Effects:** Potential on aquaculture. **Remarks**: Unsure whether all Mediterranean records are based on discarded dead specimens imported with shrimps, or whether specimens obtained from fishermen may actually originate from the Atlantic trawling by fleets based in the Mediterranean Sea; Mannino et al. [73] suggest excluding this taxon from all Mediterranean lists until confirmation of living specimens with reliable distributional data.
	**Species that spread unaimed**				
	**Species and Species ID**	**Ecoregions in South America**	**Place and Date of Entry and Reference**	**Introduction Mechanism, Pathway Category, and Subcategory**	**Impacts/Effects/Remarks**
**Gastropoda | Systellommatophora | Onchidiidae**					
	*Onchidella marginata* (Couthouy, 1852) https://www.molluscabase.org/aphia.php?p=taxdetails&id=509919, accessed on 21 April 2025	12, 13, 14, 15, 27, 28	Campbell Is., New Zealand [74]	Spread, unaimed, natural dispersal rafting on kelps	**Impacts: -** **Effects: -** **Remarks: -**
**Gastropoda | Neogastropoda | Cominellidae**					
	*Pareuthria fuscata* (Bruguière, 1789) https://www.molluscabase.org/aphia.php?p=taxdetails&id=491214, accessed on 1 April 2025	15, 16, 17, 18, 19	Campbell Is., New Zealand, Gough and Tristan da Cunha Is. [75]	Spread, unaimed, natural dispersal, rafting on kelps	**Impacts: -** **Effects: -** **Remarks: -**

**Table 2:** Freshwater mollusc species native to South America, non-native in other areas. Species and species ID following MolluscaBase [28]; place and date of entry from the first record, the localities where the species has been registered; introduction mechanism, pathway category, and pathway subcategory after Falkner et al. [33]; for ecoregions reference number, see Darrigran et al. [24] and Figure S2. UD, unspecified date.

	Species and Species ID	Ecoregions in South America	Place and Date of Entry and Reference	Introduction Mechanism, Pathway Category, and Subcategory	Impacts/Effects/Remarks
**Gastropoda | Hygrophila | Lymnaeidae**					
	*Galba cubensis* (L. Pfeiffer, 1839) https://www.molluscabase.org/aphia.php?p=taxdetails&id=724475, accessed on 21 April 2025	1–5, 12	Eastern Iberia, 2018 [76]	Movement of commodity, escape, horticulture; apparently originating from releases from horticultural facilities	**Impacts: -** **Effects:** Intermediate host (of *Fasciola hepatica* Linnaeus, 1758) **Remarks:** Non-native in anthropogenic environments, native in natural environments.
**Gastropoda | Littorinimorpha | Cochliopidae**					
	*Heleobia charruana* (d’Orbigny, 1841) https://www.molluscabase.org/aphia.php?p=taxdetails&id=760578, accessed on 14 April 2025	37–39, 41–43	Barking Creek (Thames, England, U.K.), 2003; Antwerp, Belgium, May 2014; The Netherlands, Noord Holland, North Sea Canal and surroundings [77])	Vector, stowaway, ballast water	**Impacts:** Potential displacement of native species. **Effects: -** **Remarks:** *H. charruana* is often a dominant species where it occurs.
**Gastropoda | Hygrophila| Planorbidae**					
	*Biomphalaria glabrata* (Say, 1818) https://www.molluscabase.org/aphia.php?p=taxdetails&id=848622, accessed on 13 April 2025	2, 5, 7, 8, 10, 16, 25–27, 34–36, 39, 41	Lesser Antilles, 1970–1979 (Dominica), Haıti, Dominican Republic; canals in Egypt and from the irrigation and drainage systems in the Nile Delta area, around 1981; South Africa, mid-1980s [78]	Movement of commodity, escape, research	**Impacts: -** **Effects:** Intermediate host (of *Schistosoma mansoni* Sambon, 1907). **Remarks:** *B. glabrata* was introduced into laboratories in South Africa for the maintenance of cultures *Schistosoma mansoni.* It was shown experimentally to be slightly susceptible to *S. mansoni* from Egypt and Israel. Despite several searches in South Africa, it has not been collected in recent years.
	*Biomphalaria tenagophila* (A. d’Orbigny, 1835) https://www.molluscabase.org/aphia.php?p=taxdetails&id=1001488, accessed on 1 April 2025	1–5, 7, 10, 12, 14–20, 23–27, 29, 30, 33–35, 41–45, 47	Congo, early 1970 Romania, 2004 [79,80]	Movement of commodity, escape, contaminant of plants	**Impacts: -** **Effects:** Intermediate host (of *Schistosoma mansoni* Sambon, 1907). **Remarks: -**
	*Biomphalaria straminea* (Dunker, 1848) https://www.molluscabase.org/aphia.php?p=taxdetails&id=1060816, accessed on 11 April 2025	2, 7, 10, 14–16, 21–28, 30, 33–35, 41–44, 47, 48	Costa Rica, 1976; Lesser Antilles and Martinique, approx. 1950; Grenada, 1970; Hong Kong Special Administrative Region, 1973; Shenzhen city, Guangdong province in China, 1981; Guadeloupe, 1985; St. Lucia, 1992 [80,81,82]	Movement of commodity (?), contaminant (?), contaminant of plants?	**Impacts: -** **Effects:** Intermediate host (of *Schistosoma mansoni* Sambon, 1907). **Remarks:** It is considered to be the most important intermediate host of *Schistosoma mansoni*.
**Gastropoda | Architaenioglossa | Ampullariidae**					
	*Marisa cornuarietis* (Linnaeus, 1758) https://www.molluscabase.org/aphia.php?p=taxdetails&id=737469, accessed on 20 April 2025	1, 3, 7	Cuba, 1950; St. Kitts, 1950s; Puerto Rico, 1952; near Coral Gables, Florida, USA, 1957; Egypt, 1972; Tanzania, 1977; Texas in the headwaters of the San Marcos River, San Marcos (city), Hays County, 1981; Sudan, 1981; Costa Rica, UD; Panama, UD; Dominican Republic, 1986; Martinique, 1987; Idaho, USA: Outfall of geothermally heated spring waters from a tropical fish hatchery on Deep Creek in the central Snake River drainage, Twin Falls County, 1992; California, USA, 2003; Grenada, 2009; North Spain, Colloto, Río Nora (43°22′ N-5°47′ W), 2012; Hungary, UD (only in an urban section of a stream close to the outflow from a thermal spa [83,84,85]	Movement of commodity, escape, release, ornamental, biological control [86]	**Impacts: -** **Effects:** Control (of freshwater weeds and *Biomphalaria* sp.). **Remarks:** The species alters the structure of the macrophyte community through selective herbivory, and prey on or compete with other snails [87].
	*Pomacea canaliculata* (Lamarck, 1822) https://www.molluscabase.org/aphia.php?p=taxdetails&id=741113, accessed on 20 April 2025	30, 33, 34, 41–44, 47, 48	Taiwan, 1979–1981; Philippines, 1980; Indonesia, 1981–1984; China, 1981–1985; Japan, 1981; South Korea, 1981–1986; Thailand, 1982–1990; Russia, 1986 (Kamchatka); Malaysia, 1987–1992; Vietnam, approx.. 1988; Guam, 1989; Hawaii, 1989; Singapore, 1991; South Africa, before 1991; Dominican Republic, 1991; Papua New Guinea, 1991; Laos, 1991–1994; USA (continental), 1997; Spain, 2001; Bangladesh, 2006; Cambodia, 2006; Egypt, 2006; India, 2006; Myanmar, 2008; Mexico, 2009; Iraq, 2013; Trinidad, 2014; Kenya, 2020 [85,88,89]	Movement of commodity, escape, ornamental; aquaculture	**Impacts:** Displacement of native species. **Effects:** Intermediate host (of *Angiostrongylus cantonensis* and *Gnathostoma spinigerum* [90,91]); negative on crops. **Remarks:** Possible intermediate host for parasites transmissible to humans, such as. Alters the structure of the macrophyte community through selective herbivory and food chains [87].
	*Pomacea maculata* (Perry, 1810) https://www.molluscabase.org/aphia.php?p=taxdetails&id=737473, accessed on 20 April 2025	15, 16, 20–26, 28–30, 33, 34, 42, 43	Thailand, 1990; USA, 1989; Cambodia, before 1995; China, 2006–2007; Israel, 2008; Singapore, 2008; South Korea, 2008; Malaysia, 2008; Vietnam, 2008; Japan, 2008–2013; Spain, 2009; Pakistan, 2009; Philippines, 2013 [85]	Movement of commodity, escape, aquaculture [92]	**Impacts:** On community structure (alters macrophyte and benthic communities and food chains [87]). **Effects:** Intermediate host (of trematode and human parasitic nematodes); negative on crops. **Remarks: -**
	*Pomacea diffusa* (Blume, 1957) https://www.molluscabase.org/aphia.php?p=taxdetails&id=848365, accessed on 10 April 2025	11, 13, 15, 16, 20, 21–23, 28, 29	USA (continental), 1950s; Hawaii, 1962; Sri Lanka, early 1980s; Australia, 2004; Panamá, 2008; New Zealand, 2010 [85]	Movement of commodity, escape, pet [87]	**Impacts: -** **Effects:** Food resource; aquariums (detritus and algae cleaner). **Remarks: -**
	*Pomacea scalaris* (d’Orbigny, 1835) https://www.molluscabase.org/aphia.php?p=taxdetails&id=741148, accessed on 20 April 2025	28–30, 33, 42, 43	Taiwan, 1989 [85]	Movement of commodity, escape, live food [87]	**Impacts: -** **Effects: -** **Remarks: -**

**Table 3:** Land mollusc species native to South America, non-native in other areas. Species and species ID following MolluscaBase [28]; place and date of entry from the first record, the localities where the species has been introduction mechanism, pathway category, and pathway subcategory after Falkner et al. [33]; for ecoregions reference number, see Darrigran et al. [24] and Figure S3. UD, unspecified date.

	Species and Species ID	Ecoregions in South America	Place and Date of Entry and Reference	Introduction Mechanism, Pathway Category, and Subcategory	Impacts/Effects/Remarks
**Gastropoda | Stylommatophora | Strophocheilidae**					
	*Megalobulimus oblongus* (OF Müller, 1774) https://www.molluscabase.org/aphia.php?p=taxdetails&id=1446864, accessed on 20 April 2025	1, 3, 7, 30, 32, 36, 37, 53–57, 60–66	Czech Republic, Europe, UD [93,94]; Jamaica, UD [95]	Movement of commodity, escape, ornamental	**Impacts: -** **Effects:** Negative potential on crops. **Remarks:** Species exemplifies the paradox of invaders, at risk of extinction in its native area but successfully invasive in other areas.
**Gastropoda | Stylommatophora | Bulimulinae**					
	*Bulimulus bonariensis* (Rafinesque, 1833) = *B. sporadicus* (d’Orbigny) https://www.molluscabase.org/aphia.php?p=taxdetails&id=1288732, accessed on 25 April 2025	75, 85, 87, 89, 96, 97	South States of USA, before 2009 [96]	UD	**Impacts: -** **Effects:** Negative on crops. **Remarks:** In the United States, solutions are urgently needed to manage growing populations of this species in row crops, particularly peanuts (*Arachis hipogaea* L.) and citrus. Concern in economic and food safety due to its infestation of commercial crops in southern U.S. states.
	*Bulimulus tenuissimus* (A. Férussac, 1832) https://www.molluscabase.org/aphia.php?p=taxdetails&id=1292059, accessed on 21 April 2025	28, 32, 62–67, 69, 70, 73–78, 88, 89, 92, 93	Wilmington, New Hanover County, North Carolina, USA, 1995 [97]	Movement of commodity, contaminant, contaminant of plants	**Impacts: -** **Effects:** Negative on crops; intermediate host. **Remarks: -**
**Gastropoda | Stylommatophora | Veronicellidae**					
	*Phyllocaulis gayi* (P. Fischer, 1871) https://www.molluscabase.org/aphia.php?p=taxdetails&id=1064171, accessed on 10 April 2025	101, 102	Sinaloa Mexico, 1925 [98]	Movement of commodity, escape, horticulture	**Impacts:** On native plant species. **Effects: -** **Remarks:** Naranjo et al. [99] mentioned that the record of this species in Mexico needs confirmation. In Mexico, the record for Sinaloa, nearly 100 years ago [98,100] apparently did not result in establishing breeding populations.
	*Colosius confusus* (Gomes, Robinson, Zimmerman, Obregón & Barr, 2013) https://www.molluscabase.org/aphia.php?p=taxdetails&id=1575338, accessed on 23 April 2025	12, 15, 40, 44, 48, 50	USA-Intercepted-Miami, 2001 [101]	Movement of commodity, escape, horticulture	**Impacts: -** **Effects:** Negative on crops (coffee and cultivated flowers). **Remarks: -**
	*Belocaulus angustipes* (Heynemann, 1885) https://www.molluscabase.org/aphia.php?p=taxdetails&id=1064167	75, 88–90, 97	Seven states on USA between 1960 [102], 2005 [103]; Honduras, 1984 [104]; Mexico, 1989 [105]	Movement of commodity, escape, horticulture	**Impacts: -** **Effects:** Intermediate host (of *Angiostrongylus costaricensis* Morera & Céspedes, 1971). **Remarks:** It is a synanthropic species. Recently, it was the most frequently recorded native species in Argentine nurseries [106]. Rambo et al. [107] mention it was infected with *Angiostrongylus costaricensis* Morera & Céspedes, 1971 in the municipalities of Três de Maio and Santa Rosa, in Rio Grande do Sul, Brazil, causing human abdominal angiostrongyliasis.
	*Sarasinula linguaeformis* (Semper, 1885) https://www.molluscabase.org/aphia.php?p=taxdetails&id=1441990, accessed on 20 April 2025	42, 75, 76, 90	Guadalupe Island [108]; Dominican Island; San Pedro (Intercepted by Agricultural Department, USA, lot USDA 20060114-00); Martinique (Intercepted by Agricultural Department, USA, lot USDA BX090708-006, BX090708-007)	Movement of commodity, contaminant, timber trade contaminant	**Impacts: -** **Effects:** Negative on crops (soybeans and beans). **Remarks: -**
**Gastropoda | Stylommatophora | Streptaxidae**					
	*Streptaxis contusus* (Férussac, 1821) https://www.molluscabase.org/aphia.php?p=taxdetails&id=1326821, accessed on 26 April 2025	76, Southeast and south Brazil	Hawaii, 1961 [109]	Movement of commodity, release, biological control	**Impacts: -** **Effects: -** **Remarks:** Introduced in Hawaii in 1961 as a possible biological control of the giant African snail *Lissachatina fulica* (Bowdich, 1822), but did not establish as *Streptaxis contundata*) [109].
**Gastropoda | Stylommatophora | Scolodontidae**					
	*Tamayoa decolorata* (Drouët, 1859) https://www.molluscabase.org/aphia.php?p=taxdetails&id=1442170, accessed on 27 April 2025	28	Barbados, 2001 [110]; Jamaica, 2006 Guadeloupe, Dominica, Saint Vincent [95]	UD	**Impacts: -** **Effects: -** **Remarks: -**

## Data Availability

Dataset available on request from the authors.

## Supplementary Materials

The following supporting information can be downloaded at: https://www.mdpi.com/article/10.3390/biology14111538/s1, Figure S1: South America marine ecoregions; Figure S2: South America freshwater ecoregions; Figure S3: South America land ecoregions; Table S1: Biology traits of South America native mollusc species, non-native in other continents.
