# Supplementary material for: Molluscs from South America to the World: Who and Where Are They?
Source: Biology (Basel). 2025 Nov 3;14(11):1538. doi: 10.3390/biology14111538 (PMC12650473; doi:10.3390/biology14111538)
Supplement: Supplementary file 1 [file biology-14-01538-s001.zip › Darrigran et al online resource 1.pdf]

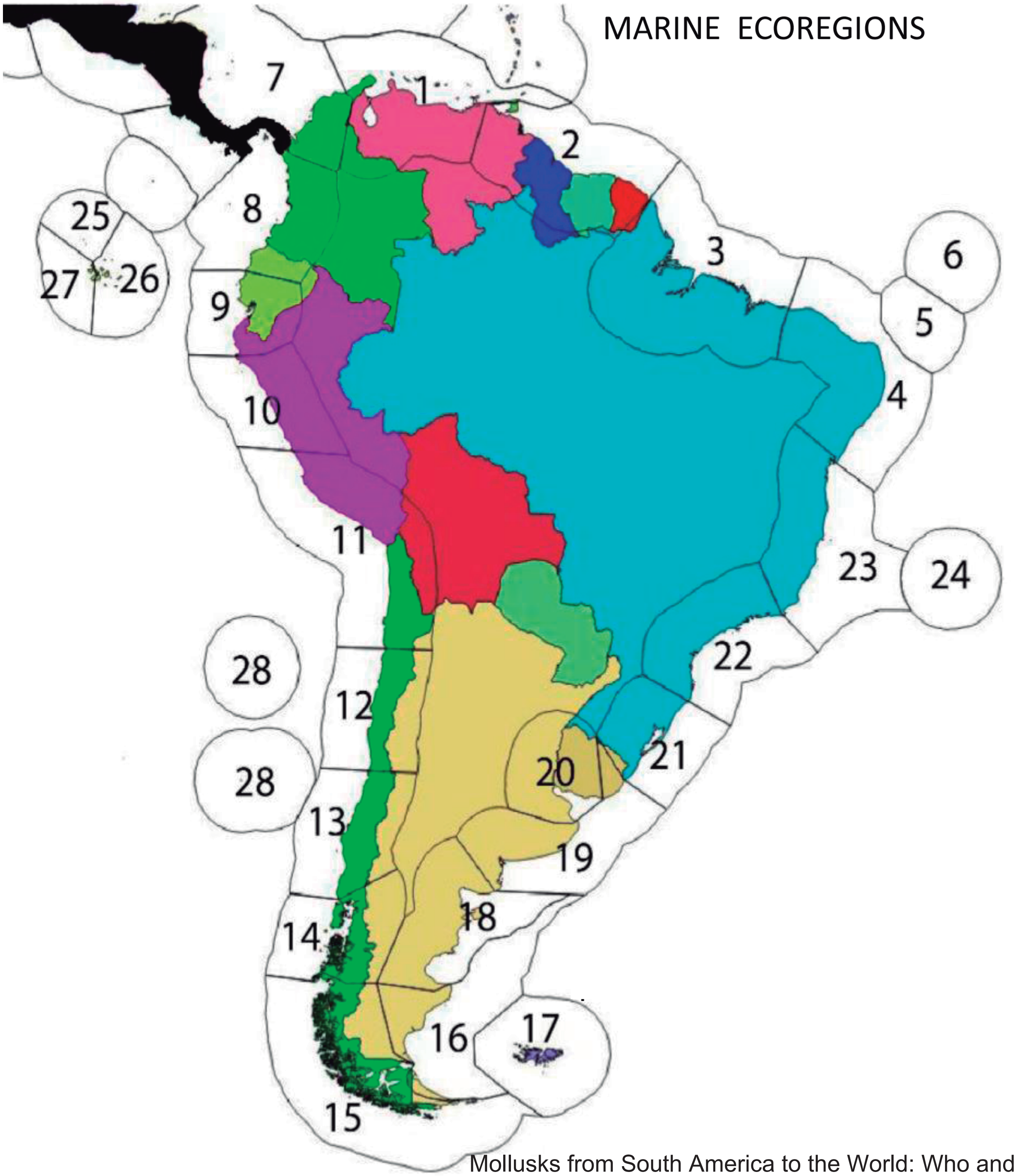

Mollusks from South America to the World: Who and where are they?

Darrigran et al.
Biology MDP

Corresponding author: C Damborenea,
Div Zool Inv, Museo de La Plata; FCNyM-UNLP- CONICET;
Paseo del Bosque, 1900 La Plata, Argentina.
cdambor@fcnym.unlp.edu.ar

28 ecoregions are recognized for marine environments [29, 30]

1. Southwestern Caribbean	8. Panama Bight	15. Channels and Fjords of Southern Chile	22. Southeastern Brazil
2. Guianan	9. Guayaquil	16. Patagonian Shelf	23. Eastern Brazil
3. Amazonia	10. Central Peru	17. Malvinas/ Falklands	24. Trindade and Martin Vaz Islands
4. Northeastern Brazil	11. Humboldtian	18. North Patagonian Gulfs	25. Northen Galapagos Islands
5. Fernando de Naroha and Atoll das Roca	12 Central Chile	19. Uruguay-Buenos Aires Shelf	26. Easthern Galapagos Islands
6. Sao Pedro and Sao Paulo Islands	13. Araucanian	20. Rio de la Plata	27. Western Galapagos Islands
7. Southern Caribbean	14. Chiloense	21. Rio Grande	28. Juan Fernandez and Desventuradas
