# Supplementary material for: Molluscs from South America to the World: Who and Where Are They?
Source: Biology (Basel). 2025 Nov 3;14(11):1538. doi: 10.3390/biology14111538 (PMC12650473; doi:10.3390/biology14111538)
Supplement: Supplementary file 1 [file biology-14-01538-s001.zip › Darrigran et al online resource 2.pdf]

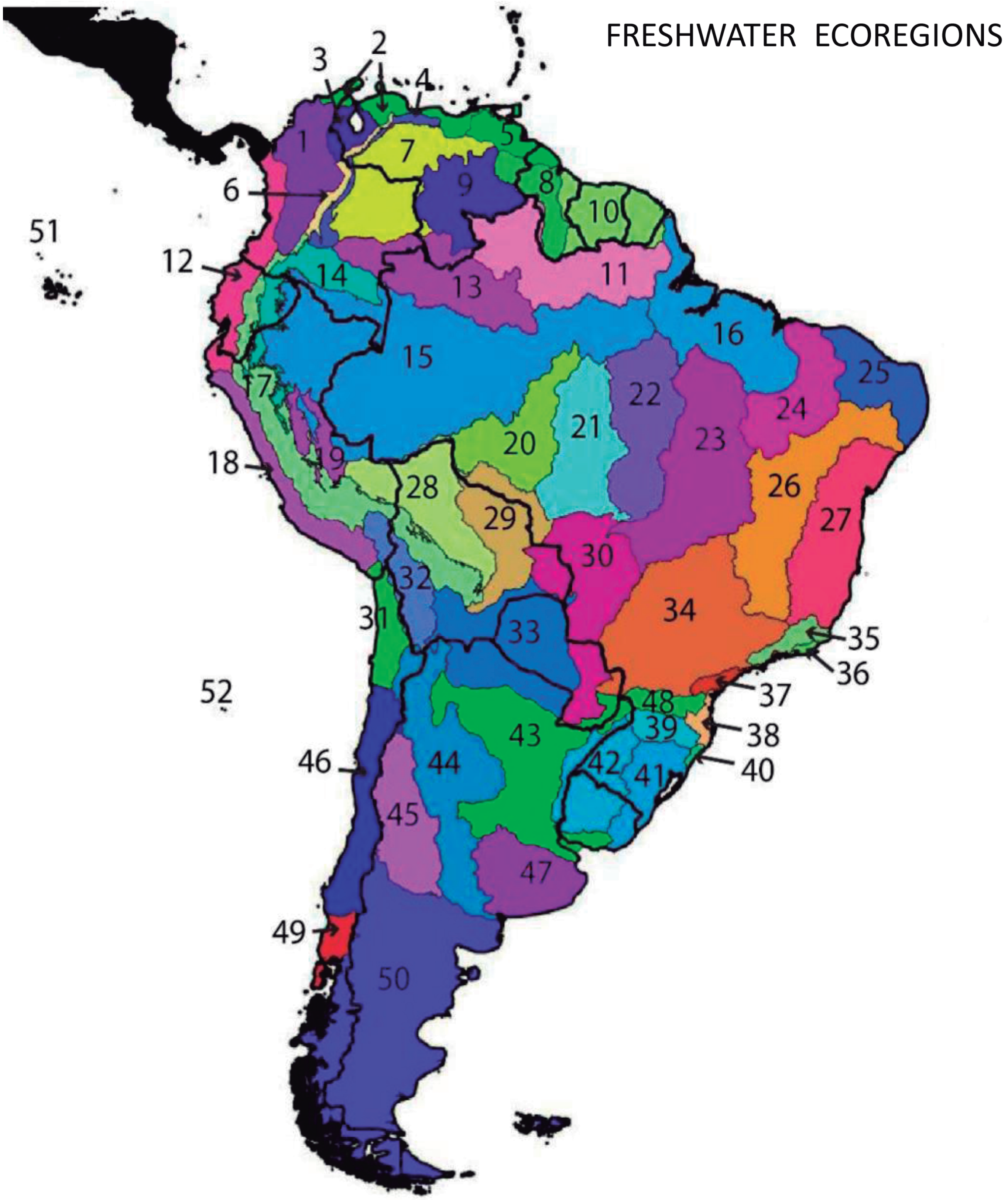

Mollusks from South America to the World: Who and where are they?

Darrigran et al.
Biology MDP

Corresponding author: C Damborenea,
Div Zool Inv, Museo de La Plata; FCNyM-UNLP- CONICET;
Paseo del Bosque, 1900 La Plata, Argentina.
cdambor@fcnym.unlp.edu.ar

52 ecoregions are recognized for freshwater environments [30, 31]

1. Magdalena - Sinu	14. Western Amazon Piedmont	27. Northeastern Caatinga & Coastal Drainages	40. Tramandi - Mampituba
2. South America Caribbean Drainages - Trinidad	15. Amazonas Lowlands	28. Mamore – Madre de Dios Piedmont	41. Laguna dos Patos
3. Maracaibo	16. Amazonas Estuary & Coastal drainages	29. Guapore - Itenez	42. Lower Uruguay
4. Orinoco Piedmont	17. Amazonas High Andes	30. Paraguay	43 Lower Parana
5. Orinoco Delta & Coastal Drainages	18. Central Andean Pacific Slopes	31. Atacama	44. Mar Chiqita – Salinas Grandes
6. Orinoco High Andes	19. Ucayali – Urubamba Piedmont	32. Titicaca	45. Cuyan - Desaguadero
7. Orinoco Llanos	20. Madeira Brazilian Shield	33. Chaco	46. South Andean Pacific Slopes
8. Essequibo	21. Tapajos – Juruena	34. Upper Parana	47. Bonaerensean Drainages
9. Orinoco Guiana Shield	22. Xingu	35. Paraiba do Sul	48. Iguassu
10. Guianas	23. Tocantins - Araguaia	36. Fluminense	49. Valdivian Lakes
11. Amazonas Guiana Shield	24. Parnaiba	37. Ribeira de Iguape	50. Patagonia
12. North Andean Pacific Slopes – Rio Atrato	25. Northeastern Mata Atlantica	38. Southeastern Mata Atlantica	51. Galapagos Islands
13. Rio Negro	26. San Francisco	39. Upper Uruguay	52. Juan Fernandez Island
