# Supplementary material for: Molluscs from South America to the World: Who and Where Are They?
Source: Biology (Basel). 2025 Nov 3;14(11):1538. doi: 10.3390/biology14111538 (PMC12650473; doi:10.3390/biology14111538)
Supplement: Supplementary file 1 [file biology-14-01538-s001.zip › Darrigran et al online resource 3.pdf]

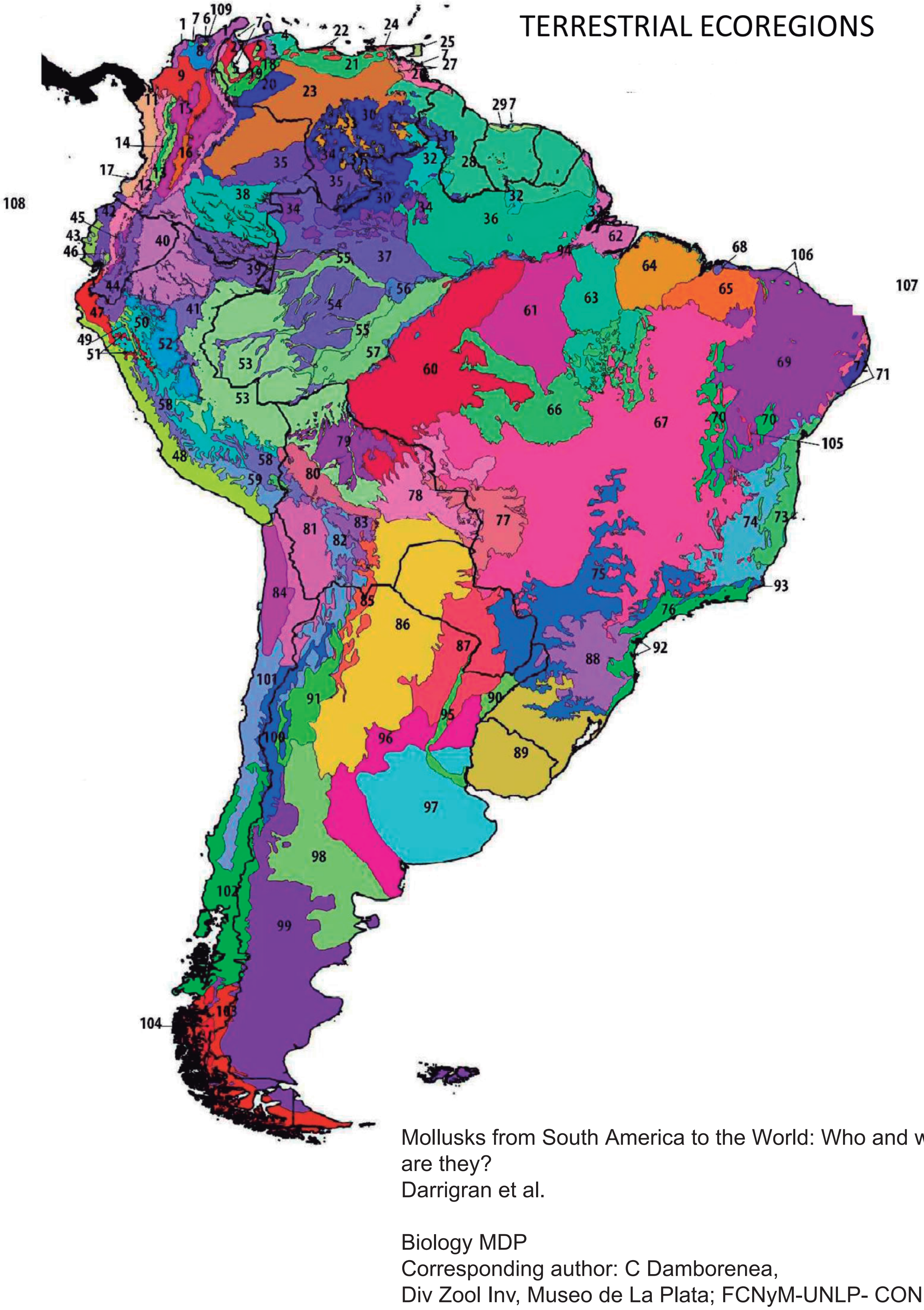

Mollusks from South America to the World: Who and where are they?

Darrigran et al.

Biology MDP
Corresponding author: C Damborenea,
Div Zool Inv, Museo de La Plata; FCNyM-UNLP- CONICET;
Paseo del Bosque, 1900 La Plata, Argentina.
cdambor@fcnym.unlp.edu.ar

109 ecoregions are recognized for terrestrial environments [29] 1/2			
1. Guajira-Barranquilla xeric scrub	22. Cordillera La Costa montane forests	43. Ecuadorian dry forests	64. Tocantins/Pindare moist forests
2. Maracaibo dry forests	23. Llanos	44. Eastern Cordillera real montane forests	65. MaranhÒo Babaþu forests
3. Paraguana xeric scrub	24. Araya and Paria xeric scrub	45. Northern Andean páramo	66. Mato Grosso seasonal forests
4. Lara-Falcón dry forests	25. Trinidad & Tobago main forest	46. Guayaquil flooded grasslands	67. Cerrado
5. Catatumbo moist forests	26. Orinoco Delta swamp forests	47. Tumbes-Piura dry forests	68. Northeastern Brazil restingas
6. Santa Marta montane forests	27. Orinoco wetlands	48. Sechura desert	69. Caatinga
7. Amazon-Orinoco-Southern Caribbean mangroves	28. Guianan moist forests	49. Marañón dry forests	70. Atlantic dry forests
8. Sinú Valley dry forests	29. Guianan freshwater swamp forests	50. Peruvian Yungas	71. Pernambuco interior forests
9. Magdalena-Urabá moist forests	30. Guianan piedmont and lowland moist forests	51. Cordillera Central páramo	72. Pernambuco coastal forests
10. Cordillera Oriental montane forests	31. Guianan Highlands moist forests	52. Ucayali moist forests	73. Bahia coastal forests
11. Chocó-Darién moist forests	32. Guianan savanna	53. Southwest Amazon moist forests	74. Bahia interior forests
12. Northwestern Andean montane forests	33. Pantepui	54. Jurußß-Purus moist forests	75. Alto Paraná Atlantic forests
13. Cauca Valley montane forests	34. Rio Negro campinarana	55. Purus varzeá	76. Serra do Mar coastal forests
14. Cauca Valley dry forests	35. Negro-Branco moist forests	56. Monte Alegre varzeá	77. Pantanal
15. Magdalena Valley montane forests	36. Uatuma-Trombetas moist forests	57. Purus-Madeira moist forests	78. Chiquitano dry forests
16. Magdalena Valley dry forests	37. Japurá-Solimoes-Negro moist forests	58. Central Andean wet puna	79. Beni savanna
17. South American Pacific mangroves	38. Caqueta moist forests	59. Central Andean puna	80. Bolivian Yungas
18. Venezuelan Andes montane forests	39. Solimoes-Japurß moist forests	60. Madeira-Tapajós moist forests	81. Central Andean dry puna
19. Cordillera de Merida pßramo	40. Napo moist forests	61. Tapajós-Xingu moist forests	82. Central Andean punan
20. Apure-Villavicencio dry forests	41. Iquitos varzeá	62. Marajó varzeá	83. Bolivian montane dry forests
21. La Costa xeric shrublands	42. Western Ecuador moist forest	63. Xingu-Tocantins-Araguaia moist forests	84. Atacama desert

109 ecoregions are recognized for terrestrial environments

85. Southern Andean Yungas	92. Southern Atlantic mangroves	99. Patagonian steppe	106. Caatinga Enclaves moist forests
86. Dry Chaco	93. Atlantic Coast restingas	100. Southern Andean steppe	107. Fernando de Noronha-Atol das Rocas moist forests
87. Humid Chaco	94. Gurupa varzeá	101. Chilean matorral	108. Galápagos Islands scrubland mosaic
88. Araucaria moist forests	95. Paranß flooded savanna	102. Valdivian temperate forests	109. Santa Marta páramo
89. Uruguayan savanna	96. Espinal	103. Magellanic subpolar forests	
90. Southern Cone Mesopotamian savanna	97. Humid Pampas	104. Rock and Ice	
91. High Monte	98. Low Monte	105. Campos Rupestres montane savanna	
